# Voluntary Medical Male Circumcision and Incident HIV Infection Among Men Who Have Sex With Men in China (The CoM Study): Protocol for a Randomized Controlled Trial

**DOI:** 10.2196/47160

**Published:** 2023-05-29

**Authors:** Yanxiao Gao, Weiran Zheng, Yinghui Sun, Luoyao Yang, Zhihui Guo, Yuwei Li, Yi-Fan Lin, Zhen Lu, Tanwei Yuan, Yuewei Zhan, Han-Zhu Qian, Bin Su, Zhiqiang Zhu, Junyi Duan, Guanghui Wang, Xin Cui, Lin Ouyang, Genshen Sheng, Yepeng Zhou, Ao Long, Yuming Yao, Thomas Fitzpatrick, Maohe Yu, Guohui Wu, Huachun Zou

**Affiliations:** 1 School of Public Health Sun Yat-sen University Shenzhen China; 2 GlaxoSmithKline plc Rockville, MD United States; 3 Beijing Youan Hospital Capital Medical University Beijing China; 4 Qingdao Qingtong AIDS Prevention Volunteer Service Center Qingdao China; 5 Chongqing Municipal Center for Disease Control and Prevention Chongqing China; 6 Shenzhen Rainbow 258 Centre for Men Shenzhen China; 7 Foshan Pengyou Care and Rescue Center for AIDS Prevention Foshan China; 8 Luzhou Red Ribbon Heart Association Luzhou China; 9 Zhitong LGBT Service Center Guangzhou China; 10 Department of Internal Medicine University of Washington Seattle, WA United States; 11 Tianjin Municipal Center for Disease Control and Prevention Tianjin China; 12 Kirby Institute University of New South Wales Sydney Australia

**Keywords:** voluntary medical male circumcision, HIV, men who have sex with men, randomized controlled trial, China

## Abstract

**Background:**

Systematic reviews and meta-analyses based on observational studies have shown voluntary medical male circumcision (VMMC) may reduce HIV risk among men who have sex with men (MSM). There is a lack of randomized controlled trial (RCT) data assessing the efficacy of VMMC.

**Objective:**

The primary objective of this study is to assess the efficacy of VMMC for preventing HIV acquisition among MSM who primarily engage in insertive anal sex.

**Methods:**

A multicenter RCT will be conducted among MSM in 8 cities in China. Eligible participants are men aged 18-49 years who self-report ≥2 male sex partners in the past 6 months, predominantly practice insertive anal sex, and are willing to undergo circumcision. Interested men who satisfy inclusion criteria will be tested for HIV 1 month before enrollment and at enrollment, and only those who are HIV negative will be enrolled. At baseline, all enrolled participants will be asked to report sociodemographic characteristics and sexual behaviors; provide a blood sample for HIV, syphilis, and herpes simplex virus type 2 testing; and provide a penile swab for human papillomavirus testing. Participants will be randomly assigned to the intervention or control group. Those in the intervention group will receive VMMC and undergo a web-based weekly follow-up assessment of postsurgery healing for 6 consecutive weeks. All participants will be tested for HIV at 3-, 6-, 9-, and 12-month follow-ups. All participants will also be asked to report sexual behaviors and undergo repeat herpes simplex virus type 2 and human papillomavirus testing at 6- and 12-month follow-ups. The primary end point is HIV seroconversion. Secondary end points are the safety and satisfaction with VMMC and the changes in sexual behaviors after VMMC. The grouped censored data will be analyzed by intention-to-treat approach.

**Results:**

Recruitment for the RCT began in August 2020 and continued through July 2022. Data collection is expected to be completed by July 2023, and full data analysis is going to be completed by September 2023.

**Conclusions:**

This study will be the first RCT to assess the efficacy of VMMC in preventing HIV infection among MSM. Results from this trial will provide preliminary evidence for the potential efficacy of VMMC to reduce incident HIV infection among MSM.

**Trial Registration:**

Chinese Clinical Trial Registry ChiCTR2000039436; https://www.chictr.org.cn/showproj.html?proj=63369

**International Registered Report Identifier (IRRID):**

DERR1-10.2196/47160

## Introduction

### Overview

Men who have sex with men (MSM) are at high risk for HIV infection. Evidence-based HIV preventive measures for MSM include behavioral interventions (eg, condom use) and biomedical interventions (eg, preexposure prophylaxis [PrEP] and postexposure prophylaxis [PEP]). Condoms are highly effective in preventing HIV transmission, but the prevalence of condomless sex remains high among MSM [1,2]. HIV PrEP reduces risk of HIV infection, but uptake among MSM is limited [3]. The effectiveness of HIV PEP is constrained by lack of awareness, limited availability of PEP services, and poor adherence to medications and clinic follow-ups, particularly in resource-limited settings [4-6]. Innovative, effective, and acceptable HIV prevention measures are needed to complement and expand existing prevention strategies among MSM.

Voluntary medical male circumcision (VMMC) may protect men from HIV infection by reducing mucosal surface area and the concentration of HIV target cells in the penis [7,8]. Three large randomized controlled trials (RCTs) in Africa found that VMMC reduced the risk of HIV infection by 50%-60% among heterosexual men [9-11]. Debate remains on whether VMMC can effectively prevent HIV acquisition among MSM. Two systematic reviews and meta-analyses in 2008 and 2011 [12,13] showed that there was no statistically significant association between VMMC and HIV acquisition among MSM. However, new evidence has emerged over the past 10 years. A 2018 meta-analysis found that VMMC may reduce risk of HIV infection by 20% among MSM [14]. Similarly, a 2019 systematic review and meta-analysis of 67 observational studies [15] reported that VMMC was associated with a 23% lower odds of HIV infection among MSM overall and 42% lower odds of HIV among MSM in low- and middle-income countries. Furthermore, VMMC was associated with a 16% lower odds of herpes simplex virus and a 29% lower odds of penile human papillomavirus (HPV) infection among MSM living with HIV [15]. Another systematic review in 2019 [16] found VMMC was associated with 7% and 38% lower odds of HIV infection among MSM overall and MSM in Asia and Africa, respectively. These systematic reviews found that no previous RCTs have evaluated the impact of circumcision on HIV acquisition among MSM. Interventional trials are needed to confirm the findings of previous observational studies.

Sexual position preference may have a significant impact on the efficacy of VMMC to reduce HIV infection among MSM. Men who practice insertive anal sex may benefit more from VMMC than those who practice receptive anal sex. Subanalysis in a 2019 systematic review and meta-analysis [15] found that MSM who primarily engage in insertive anal sex had a 56% lower odds of HIV infection, a stronger association compared to what was found among MSM overall. In China, 70% of MSM practice either exclusively insertive anal sex or both insertive and receptive anal sex [17,18]. Thus, VMMC has the potential to become an important HIV prevention option for most MSM in China.

Only 15.3% of MSM in China have undergone circumcision [19]. This proportion is lower than the global average of 37%-39% [20]. Mainstream social conventions and religious practice in China are neutral on circumcision, neither promoting nor forbidding the procedure. Implementing a VMMC intervention in China should not be significantly hindered by Chinese traditions or cultural values. Other potentially effective interventions, such as PrEP, are still in promotion phase in China, which is impeded by high cost, low accessibility, and stigma among MSM [21,22]. As for consistent condom use, the rate of its use among MSM remains relatively low, approximately 50% [23,24]. Hence, it is essential to determine an alternative HIV prevention strategy in China. Given the high proportion of Chinese MSM who are uncircumcised and the need for alternative HIV prevention strategies in China, it is theoretically feasible to conduct an RCT to evaluate the efficacy of VMMC to reduce incident HIV infection among MSM in China. This RCT could provide valuable information to inform HIV prevention policy as well as future mathematical models and additional clinical trials of VMMC.

### Objectives

The primary objective of this study is to assess the efficacy of VMMC for preventing HIV acquisition among MSM who primarily engage in insertive anal sex. The secondary objectives are to assess (1) the safety of and satisfaction with surgical VMMC among MSM; (2) changes in reported sexual risk behaviors after circumcision among MSM; and (3) the efficacy of VMMC for preventing sexually transmitted infections (STIs), including herpes simplex virus type 2 (HSV-2), syphilis, and penile HPV among MSM. Our hypothesis is that VMMC could reduce the risk of HIV infection among MSM.

## Methods

### Study Design

**Figure 1:**
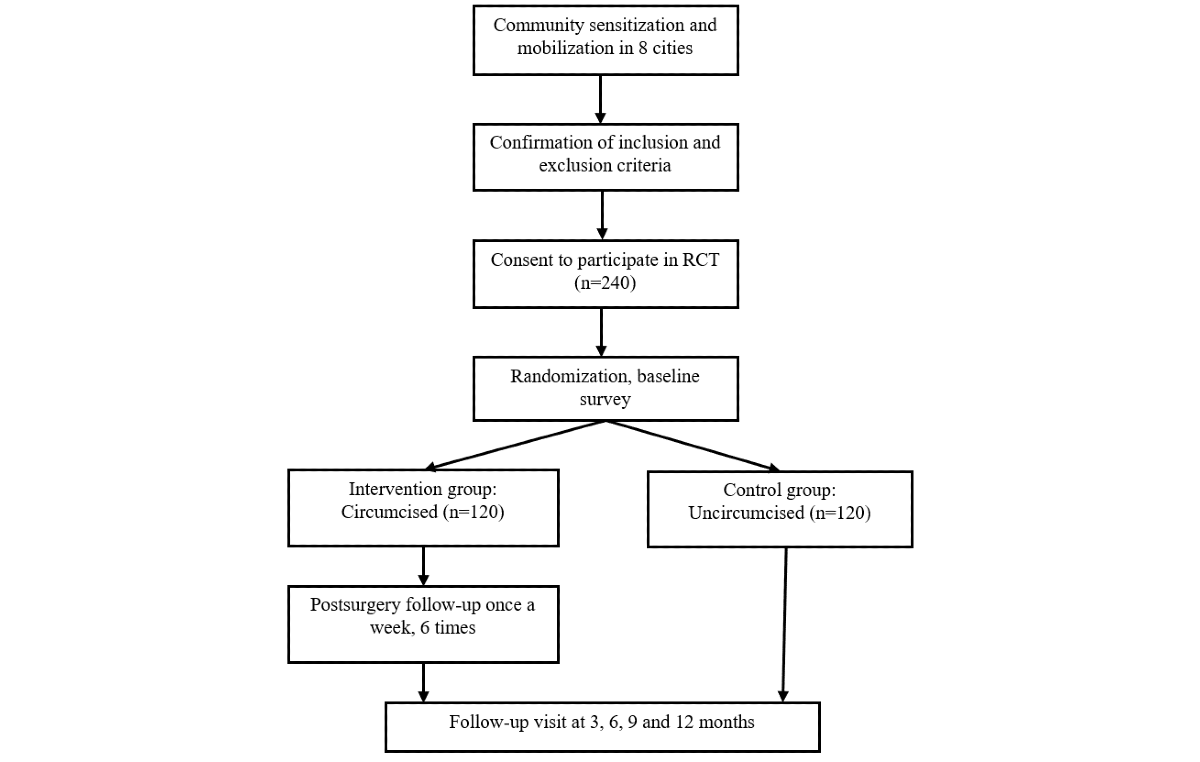
The flow chat of the CoM (circumcision of men who have sex with men) Study. RCT: randomized controlled trial.

This is a multicenter RCT to evaluate the efficacy of VMMC on incident HIV infection among MSM in China over a 12-month follow-up period (Figure 1).

### Study Setting

The VMMC and incident HIV infection among MSM in China (circumcision of men who have sex with men [CoM] Study) will be conducted in 8 cities in Mainland China: Beijing, Tianjin, Qingdao, Chongqing, Shenzhen, Guangzhou, Foshan, and Luzhou (Table 1).

This study began on August 1, 2020, and is scheduled to be completed by September 31, 2023. The study has 3 phases: eligibility screening, enrollment, and follow-up (Table 2).

**Table 1:** Study sites of the CoM (circumcision of men who have sex with men) Study.

City and province	Study site	Nature of study site
Beijing	Beijing You’an Hospital, Capital Medical University	Tertiary hospital
Tianjin	Tianjin Centers for Disease Control and Prevention	Provincial CDC^a^
Qingdao, Shangdong	Qingtong Volunteer Service Center for HIV/AIDS Prevention	NGO^b^
Guangzhou, Guangdong	Guangzhou Zhitong Public Service Center	NGO
Shenzhen, Guangdong	Shenzhen Rainbow 258 Center for Men	NGO
Foshan, Guangdong	Foshan Pengyou Care and Rescue Center for AIDS Prevention	NGO
Chongqing	AIDS/STD^c^ Control and Prevention Institute, Chongqing CDC	Provincial CDC
Luzhou, Sichuan	Luzhou Red Ribbon Heart Association	NGO

^a^CDC: centers for disease control and prevention.

^b^NGO: nongovernmental organization.

^c^STD: sexually transmitted disease.

**Table 2:** Schedule of events of the CoM (circumcision of men who have sex with men) study.

Events		Eligibility screening	Enrollment	Follow-up			
		Month 1	Month 0 (baseline)	Month 3	Month 6	Month 9	Month 12
**Recruitment and enrollment**							
	Screening questionnaire	**√**					
	One-on-one consultation	**√**					
	Watching enrollment videos	**√**					
	Informed consent		**√**				
	Assignment of study group		**√**				
	Intervention^a^		**√**				
**Assessments**							
	HIV rapid testing	**√**	**√**	**√**	**√**	**√**	**√**
	Baseline questionnaire		**√**				
	Follow-up questionnaire				**√**		**√**
	STIs screening^b^		**√**		**√**		**√**

^a^For participants in the intervention group, they will be formally enrolled after completing the circumcision. There is a maximum waiting period of 3 months for surgery and a 6-week postoperative recovery period before formal follow-up phase. For participants in the control group, they will remain uncircumcised at enrollment and follow-up, and they will enter the formal follow-up phase after completing the enrollment and baseline questionnaire.

^b^STIs include herpes simplex virus type 2, syphilis, and penile human papillomavirus.

### Interventions

VMMC is the intervention to be evaluated in this study. Men in the intervention group will undergo VMMC within 3 months of randomization, and they could choose one of 2 types of circumcision (disposable circumcision stapler surgery and conventional surgical procedure), while those in the control group will remain uncircumcised during the follow-up. All men will be followed for 12 months.

### Study Outcomes

The primary outcome is HIV incidence, comparing the intervention and control groups. Secondary outcomes are the safety and satisfaction with VMMC; changes in sexual behaviors after VMMC will also be evaluated. The penile HPV incidence, clearance, and persistent infection; HSV-2 incidence; and syphilis incidence are compared in both groups.

### Eligibility Criteria

Individuals who meet all of the following criteria are eligible for enrolment in the CoM Study: (1) aged 18-49 years; (2) assigned male sex at birth; (3) ≥2 male sex partners and ≥10 episodes of anal sex in the past 6 months; (4) practiced insertive role in ≥7 of the last 10 episodes of anal sex with male sex partners; (5) HIV negative; (6) has foreskin that covers over half the glans when the penis is not erectile; (7) is willing to undergo VMMC; (8) resides in the city where a study site is located and has no plan to relocate in the next 12 months; (9) is willing to provide a blood sample and exfoliated penile swabs to test for HIV, HSV-2, syphilis, and HPV; and (10) is able to provide informed consent. Men who are already circumcised or enrolled in other biomedical HIV intervention studies will be excluded. Men who have been diagnosed with a bleeding disorder or have other medical contraindications to VMMC will also be excluded.

### Patient and Public Involvement

There was collaboration with MSM community–based organizations staff who were not involved in the study, to ensure study processes were well integrated into these settings. Patients were not involved in the design of the study.

### Participant Recruitment and Enrollment

As the distribution of our target population unknown and MSM in China are a hard-to-reach population, we will use convenience sampling and snowball sampling to recruit participants. A public account on WeChat (a multi-purpose instant messaging app developed by Tencent, with over 1 billion active monthly users) will be used to communicate with participants. Recruitment, enrollment, provision of health education, and participant follow-up will be accomplished through WeChat. This RCT will also recruit and enroll potential participants in collaboration with local MSM community–based organizations by using their organizational WeChat accounts and in-person outreach activities. An offline survey will be carried out at MSM community–based organizations and the department of dermatology at participating hospitals.

A QR code which links to an online screening questionnaire will be included in recruitment materials. Recruitment materials will include paper materials (eg, posters, folding pages, table tent cards, and business cards) and video materials (ie, a 2-minute enrollment video with a brief introduction and a 15-minute enrollment video with detailed information). Paper and video materials will include summaries of the following: (1) current state of research on efficacy of VMMC to prevent HIV infection; (2) potential benefits of VMMC; and (3) potential risks of VMMC. Potential participants will be invited to scan the QR code included in these paper or video materials and then complete a web-based screening questionnaire with preprogramed logic to assess eligibility.

Eligible men will be invited to participate in this study. Men who agree to participate will receive a face-to-face introduction to the CoM Study by a trained investigator at the study site. Questions about the study will be answered by these investigators during these introductions. For men who are willing to undergo VMMC, HIV rapid testing will be conducted 1 month before enrollment and at enrollment.

### Randomization and Follow-Up

Eligible men will be randomized using a 1:1 allocation ratio to the intervention and control groups. Randomization lists for each study site have been pregenerated by researchers. Each envelope with a unique number will be assigned for 1 allocation sequence, into which a card with grouping information will be put before sealing. Trained investigators at each study site will assign an envelope to each participant according to the order of the participant’s arrival. Participants cannot be blinded due to the nature of the intervention. After randomization, trained researchers will provide HIV prevention counseling to participants in both groups, including promotion of consistent condom use and regular HIV testing.

Participants randomized to the intervention group will be encouraged to complete VMMC within 1 week of enrollment. Time to VMMC can be extended up to 3 months after enrollment on a case-by-case basis. We recommend participants to undergo VMMC at a secondary or higher-level medical center of their own choice. Although the clinical VMMC activities are not part of study procedures, a research assistant would accompany the participants to the hospital to undergo the circumcision. This trial will provide a surgical subsidy of CNY 1000 (US $160). If participants do not complete VMMC within 1 month of enrollment, a text message reminder will be sent. The surgeon will check the condition of the foreskin of participants before the operation to determine if surgery is necessary. Men in the intervention group will be advised to abstain from sex for 6 weeks after undergoing VMMC, and the 12-month follow-up period will begin after this 6-week period of abstinence. If men resume sexual behaviors earlier than 6 weeks after VMMC, then the 12-month follow-up period will begin at the time of their first sexual encounter after VMMC.

Men randomized to the control group will complete the baseline survey after enrollment. The 12-month follow-up period for the control group will begin immediately after completing the baseline survey. At the end of the 12-month follow up period, men in the control group will be offered a CNY 1000 (US $160) subsidy to undergo VMMC.

### Study Measures

The baseline and follow-up surveys will collect information on sociodemographic characteristics (including age, ethnic, educational background, marital status, and monthly individual income) and sexual behaviors (including the number of male and female sex partners, frequency of vaginal or anal sex, condom use, and HIV-positive sex partners) for all enrollment participants. Follow-up surveys will be completed at 6- and 12-month follow-ups. Men randomized to receive VMMC will also be asked to complete weekly follow-up surveys for 6 weeks after VMMC to monitor for postsurgical complications, including pain, poor surgical site wound healing or infection, and premature resumption of sexual behaviors. If an adverse event is reported, trained investigators will contact participants and provide advice on whether further clinical evaluation is needed.

Fingertip blood drop samples will be collected at baseline as well as 3-, 6-, 9- and 12-month follow-ups and tested for HIV using an HIV rapid test. Venous blood samples collected at baseline as well as 6- and 12-month follow-ups will be tested for HIV, syphilis, and HSV-2. Penile swab samples will be collected at baseline as well as 6- and 12-month follow-ups and tested for HPV.

HIV rapid testing will detect antibodies against HIV-specific antigens using a solid-phase immunochromatographic assay (SD Bioline HIV/Syphilis Duo test; Standard Diagnostics Inc), and these assays will be performed at study sites. Venous blood samples will be tested for HIV using a chemiluminescent microparticle immunoassay (Anti-HIV CLIA Microparticles; Autobio Diagnostics Co) performed at Guangzhou Hybribio Medical Laboratory Limited. If a participant’s sample is found to have a positive result for HIV, we will recollect a venous blood sample from that participant and perform a confirmatory Western blot test at the local Centers for Disease Control and Prevention. Except for HIV, laboratory assays will be applied for syphilis, HSV-2, and HPV test. HPV DNA testing and genotyping was performed using the 37 HPV GenoArray Diagnostic Kit (Hybribio; Chaozhou, China). Syphilis serological tests will be performed using tolulized red unheated serum test and treponema pallidum particle assay. Detection of HSV-2 immunoglobulin G and HSV-2 immunoglobulin M in blood samples are based on a chemiluminescent microparticle immunoassay. Participants who test positive for pathogens or antibodies will be offered STI counseling and referred to a hospital for treatment.

### Participation Incentives and Follow-Up Reminders

Web-based educational materials on sexual health will be regularly posted for all participants to read via WeChat by the CoM Study’s official WeChat account. Short voluntary quizzes will be included at the end of each posted educational material, and participants who complete these quizzes will receive a CNY 2 or CNY 5 (US $0.3 or US $0.8) reimbursement. Each participant will also receive a CNY 50 (US $8) reimbursement each time they complete a questionnaire and provide a blood sample for HIV and STI testing. Participants who complete the surgery according to the study procedures will receive CNY 1000 (US $160) surgery subsidy. In addition, participants will receive an additional CNY 500 (US $80) subsidy after completing all required follow-up surveys as well as HIV and STI testing.

Reminders to complete follow-up surveys will be sent to all enrolled men via WeChat. If follow-up surveys are not completed on time, investigators will contact enrolled participants with additional reminders each week for up to 5 weeks to encourage participation and minimize loss to follow-up.

### Data Collection and Management

All data will be collected using Wenjuanxing (Ranxing Information Technology Co, Ltd [25]), a web-based questionnaire platform. Several quality-control questions will be included in the study questionnaires, and the consistency of answers will be examined by trained investigators. Information concerning identification, eligibility, baseline surveys, intervention, and follow-up surveys will be recorded in case record form. Adverse events will also be recorded. Identity information will be collected for allowance distribution purpose only, which will not be used for statistical analysis or intended for information leakage. Only study staff will be able access the study database, which will be protected with a unique log-in password that will be updated monthly to maximize data safety. The data safety monitoring committee (DSMC) of the CoM Study will be comprised of 2 statisticians, 2 clinicians, and key study investigators. The DSMC will supervise procedures, including data quality control, data integrity, data security, data sharing, and data analysis. All investigators have completed standard operating procedure training and data management training.

### Sample Size

The sample size is calculated based on the supposed protective rate of circumcision. Studies have shown that HIV incidence among MSM was 12.5/100 person-years in Chongqing [26], 15.5/100 [27] and 10.3/100 person-years [28] in Shenyang, and 15.6/100 person-years in 8 cities in China [29]. Considering participants in the CoM Study predominantly practiced insertive anal sex, consequently with lower HIV infection risk compared with those who primarily practiced receptive role, and that the effect of VMMC may be higher among men who primarily or exclusively practice the insertive anal intercourse than MSM overall, we suppose HIV incidence among MSM to be 10/100 person-years and protective rate of circumcision to be 80%, and thus, HIV incidence would be 2/100 person-years in the intervention group. A target sample size of 240 (120 in each group) is set to achieve 80% power at 1-sided α=.10, with 10% lost to follow-up.

### Statistical Analysis

All data will be analyzed based on the principle of intention to treat. We will assess the type of missing data and assess attrition effects by testing whether there are systematic differences between dropouts and nondropouts. Key baseline characteristics will be examined between the intervention and control groups to ensure they are balanced; if not, subsequent analyses will assess whether these characteristics are confounding factors of the intervention.

For continuous variables, we will calculate means and SDs, and means will be compared with 2-sample *t* tests or Mann-Whitney tests. For categorical variables, proportions will be compared with chi-square tests or Fisher exact tests. In addition, we assume that HIV status at the end of follow-ups is grouped censored data and suitable for exponential, proportional hazards model with constant baseline hazard. Therefore, differences between the intervention and the control groups will be identified with a Poisson regression model, which includes observed covariates (ie, HIV risk behaviors, such as condom use, number of sexual partners, frequency of insertive anal sex, and HIV-positive sexual partners as well as demographic variables, such as age, education level, and income). Incidence rates will be calculated for each outcome in the 2 groups. Moreover, the incidence rate ratios and their 95% CIs will be estimated using the Poisson regression model. For all the outcome variables, including the primary (ie, HIV incidence) and secondary (ie, penile HPV incidence, clearance and persistent infection, HSV-2 incidence, and syphilis incidence) outcomes of interest, the aforementioned analyses will be repeated across all the visits (ie, 6- and 12-months follow-ups).

The changes in sexual behaviors after VMMC over time between the intervention and the control groups will be compared with the generalized estimating equations. For outcomes of VMMC safety and satisfaction among circumcised MSM, we will perform the same statistical description and comparative analyses described as above.

A 2-sided *P* value lower than .05 will be used to infer statistically significant difference. All the statistical analyses will be performed with R software (version 4.2.1; R Core Team).

### Ethics Approval

This study has obtained ethics approval from Ethical Review Committee for Biomedical Research of School of Public Health (Shenzhen), Sun Yat-sen University (SYSU-PHS-2020041) and Ethics Committee of Beijing Youan Hospital, Capital Medical University (2020-049). All participants will provide written consent prior to taking part in the study.

## Results

Participant recruitment began in August 2020 and continued through July 2022. We expect that follow-up phase and data collection will be completed by July 2023, and primary data analyses will be completed by September 2023. The study findings will be submitted for presentation at relevant international conferences and for publication in a peer-reviewed journal.

## Discussion

### Principal Findings

The CoM Study is an RCT to evaluate the efficacy of VMMC to prevent incident HIV infection among MSM in China. MSM who primarily practice insertive anal sex and are willing to undergo VMMC will be recruited using promotional materials disseminated through web-based platforms and community-based organizations. Participants will be randomly assigned to receive or not receive VMMC and then will be followed for a 12-month period to assess incident HIV infection. As an RCT with limited observed person-years, priority will be given to MSM with high-risk sexual behaviors. Follow-up for MSM randomized to receive VMMC will start after a 6-week postsurgical follow-up period to ensure men in the intervention and control groups have comparable opportunity to acquire HIV after enrollment. HIV testing will be conducted every 3 months; it helps to detect acute HIV infection. Findings from this trial will provide preliminary RCT evidence on whether VMMC can effectively prevent HIV among MSM.

### Limitations

The CoM Study will have several limitations. First, because the 8 study sites are located in different parts of Mainland China, we cannot ensure that VMMC will be performed in a uniform manner for all men randomized to the intervention group. We will provide men in the intervention group with a list of hospitals that provide standard VMMC services. But men will be able to choose a hospital at their own discretion. Second, MSM who undergo VMMC may experience surgical site complications, particularly when they resume insertive anal sex. Men who have recently undergone VMMC may therefore chose to avoid insertive anal sex. This may increase their frequency of receptive anal sex, and thereby, increase their risk of HIV infection. At enrollment, participants will be provided with detailed sexual health consultations to reduce sexual intercourse during abstinence. Third, sexual health behaviors will be assessed by self-report, and therefore, may be affected by recall and social desirability biases.

### Conclusions

To achieve the sustainable development goal of ending the AIDS epidemic as a public health threat by 2030, more viable HIV prevention strategies are urgently needed. The CoM Study proposes VMMC as an intervention and assesses its preliminary efficacy to prevent incident HIV among MSM. Results from this study are expected to offer reliable data in supporting the development of mathematical models, which aim to estimate the effect of VMMC on HIV risk reduction among MSM under various conditions and assumptions.

## Abbreviations

CoM: circumcision of men who have sex with men
DSMC: data safety monitoring committee
HPV: human papillomavirus
HSV-2: herpes simplex virus type 2
MSM: men who have sex with men
PEP: postexposure prophylaxis
PrEP: preexposure prophylaxis
RCT: randomized controlled trial
STD: sexually transmitted disease
STI: sexually transmitted infection
VMMC: voluntary medical male circumcision

## Data Availability

The data sets used and analyzed during this study are available from the corresponding author on reasonable request.
